# Serum Extracellular Vesicles Reveal Metabolic Responses to Time-Restricted Feeding in High Fat Diet-Induced Obesity in Male Mice

**DOI:** 10.21203/rs.3.rs-4745029/v1

**Published:** 2024-09-24

**Authors:** Xiaoli Chen, Theresa Bushman, Te-Yueh Lin, Qin Fu, Sheng Zhang

**Affiliations:** University of Minnesota; University of Minnesota; University of Minnesota; Cornell University; Cornell University

**Keywords:** extracellular vesicles, proteomics, obesity, time-restricted feeding, inflammation

## Abstract

**Objective:**

Extracellular vesicle (EV) secretion and cargo composition are dysregulated in metabolic diseases. This study aimed to identify changes in the EV size profile and protein cargoes in diet-induced obesity following time-restricted feeding (TRF) and to establish the role of EVs in obesity-related metabolic responses.

**Methods:**

Mice were fed a high-fat diet (HFD) for 18 weeks prior to being placed either ad libitum or a time-restricted feeding for an additional 10 weeks. Mice on a normal chow ad libitum served as the control. The TRF group had food available for 10 hours and fasted for 14 hours per day.

**Results:**

The serum EV size profile and amount displayed sex- and age-dependent changes in HFD-induced obesity, with age reducing EV amounts. HFD decreased small EV populations and increased larger EV populations, while TRF reversed these changes. Quantitative proteomic analysis showed that the abundance and composition of EV proteins changed in response to both acute stimulation with lipopolysaccharides (LPS) and HFD. Gene ontology analysis identified specific sets of EV proteins and their involved biological processes, reflecting the effect of LPS and HFD, as well as the reversal effect of TRF on metabolic and inflammatory pathways. EV proteins altered by HFD and those reversed by TRF had low protein overlap but significant functional overlap in biological processes. TRF activated the PPAR signaling pathway and the AKT-mTOR signaling pathway. The most significant impacts of HFD and TRF were observed on lipoprotein and carbohydrate metabolism, complement system, and neutrophil degranulation. The reversal effect of TRF on the complement system was pathway-specific, significantly activating the lectin complement pathway and restoring neutrophil degranulation.

**Conclusion:**

Our data indicate that EVs are involved in diet-induced metabolic and inflammatory responses. Different EV populations may carry distinct sets of proteins involved in specific biological processes, thereby regulating diverse metabolic pathways efficiently.

## INTRODUCTION

In the past decade, the importance of extracellular vesicles (EVs) in cell-to-cell communication and their physiological role in metabolism has led to increased research focus on identifying their impact in obesity and diabetes. EVs have been found in various human biofluids, including plasma, saliva, breast milk, and bile ^1–3^. Tissues such as adipose tissue, liver, muscle, and pancreas have the ability to secrete EVs ^4^. Cells release different types of EVs, which can be classified based on their biogenesis, density, size, cargo, and surface molecules, reflecting the physiological state of the releasing cells ^5–7^. With carrying their various molecules, once released, EVs can interact with neighboring cells and transfer their cargo between cells, inducing changes within recipient cells ^8–10^. Cell-to-cell communication mediated by EVs can alter cellular processes, such as immune response, regulation of tissue homeostasis, and cell differentiation ^11–13^.

According to their biogenesis, EVs are classified into apoptotic bodies, microvesicles (MVs), and exosomes. MVs are formed by the direct outward budding of the plasma membrane, whereas the formation of exosomes begins within the endosomal system via the inward budding of the late endosome lumen, leading to the formation of multivesicular body (MVB) and released by fusion with the plasma membrane ^14,15^. Exosomes were initially discovered in the early 1980s and are classified within the range of 30–150nm, containing proteins, miRNAs, nucleic acids, and metabolites ^1,15,16^. The cargo or composition of EVs reflects the physiological and pathological state of the releasing cells, suggesting the composition can serve as a potential biomarker for early disease detection and monitoring ^6^.

Previous studies have demonstrated that diet, age, and obesity can affect the composition of EVs. For example, mice fed a high fat diet (HFD) exhibited change/increase in adipose tissue-derived EVs ^5,17^. In addition, research has explored the impact of caloric restriction on EV production and composition, suggesting that alterations in EV-associated proteins and miRNA may contribute to the health benefits observed from caloric restriction ^18^. EV-miRNAs have been used as a treatment to alleviate insulin resistance and glucose intolerance in mice with obesity ^19^. Age and cellular senescence have also shown changes in the amount and composition of EVs being released ^5^. In terms of EV concentration and diameter sizes, there have been reports that serum EV concentration decreases with age in male rats, along with a larger average EV diameter size ^20^. However, Alibhai et al. revealed that plasma EVs from older female mice had a smaller EV size than the young female mice ^21^. However, the investigation into sex- and age-difference in EVs size and distribution remains limited.

High-fat diet can induce obesity, insulin resistance, and dyslipidemia ^22^, as well as alter lipid metabolism in adipose tissue ^23^. Additionally, implementing time-restricted feeding (TRF), a form of intermittent fasting that limits the daily time window of energy intake, prevents HFD-induced weight gain and alterations in metabolic processes. Understanding the size, composition, and secretion mechanisms of EVs is crucial as they reflect the physiological state of the cells releasing them. Therefore, the aim of this study was to examine the impact of HFD and TRF on serum EV composition and secretion, focusing on sex and age differences. Our findings suggest that EV profile and protein changes can reflect metabolic responses to diet, and we have identified specific responsive EV proteins and their regulation of biological processes.

## METHODS

### Animal Study

Animals were housed at 22 °C in a specific pathogen-free facility at the University of Minnesota. Animal studies were conducted with the approval of the University of Minnesota Animal Care and Use Committee and conformed to the National Institute of Health guidelines for laboratory animal care (IACUC 2102A38852). The study consisted of eighteen 3-month-old C57BL/6 male mice, twelve 9–10-month-old (middle-aged) C57BL/6 female mice, and eighteen 17–21-month-old female mice (The Jackson Laboratory, Bar Harbor, ME, USA). A group of mice were fed a high-fat diet (HFD) for 18 weeks to induce obesity while 4 served as the control on a normal chow diet. Following the 18 weeks, the mice were placed into one of three groups: normal chow ad libitum (Control), high-fat diet ad libitum (HFD-AL), and HFD with time-restricted feeding (HFD-TRF). The AL groups had access to food and the TRF group had access to food 10 h/day. The mice were housed in groups of 3–4 per cage, with water ad libitum and in 12 h light/dark cycles. The high-fat diet provided to the mice was a 60% HFD (Bio-Serv: F3282) and the normal chow was provided by the animal facility (Envigo: 2918). All three experimental groups were regulated by being moved between cages with and without food. All mice were moved at 8:30 PM and 6:30 AM daily for 10 weeks during the TRF portion of the study. Food intake was measured daily by cage and divided by the number of mice within the cage. After 10 weeks of the dietary intervention, all three groups were sacrificed following a 16 h fast. Blood was collected through cardiac puncture for exosome isolation.

### Extracellular Vesicle Isolation

Exosomes were isolated from mouse serum by Total Exosome Isolation Reagent for Serum (ThermoFisher #4478360) following the manufacturer’s instructions. 20µL of reagent was added to 100 µL of serum, incubated at 4°C for 30 minutes, centrifuged at *10,000* x *g* for 10 minutes at room temperature. After centrifugation, the supernatant was aspirated and discarded. The pellet of exosomes was re-suspended in 50 µL of PBS, which contained a protease inhibitor (Roche 11697498001).

### Nanoparticle Tracking Analysis (NTA)

The Nanosight LM-10 instrument used at the Minnesota Nano Center was used to measure and visualize particle sizes and concentration of EVs isolated from mouse serum samples. Five repeated measurements of 60s were taken per sample and the mean value was used to determine diameter and particle number. NTA software measured size distribution (ranging from 10–1000nm) and concentration (particles/mL) of nanoparticles present within EV samples.

### Functional Enrichment Analysis

Proteins with a *p*-value < 0.05 between groups were recognized as significantly differentially expressed proteins. Gene Ontology (GO) analysis was conducted using Metascape (http://metascape.org) ^24^. GSEA v.4.3.3 was used for Gene Set Enrichment Analysis (GSEA) based on protein expression ^25,26^.

### Statistical Analysis

Results were expressed as mean ± SEM. Data was analyzed by student t-test, one-way ANOVA, and two-way ANOVA via GraphPad Prism (version Prism 10.1.1). *p*-values less than 0.05 were considered to be significant.

## RESULTS

### Sex- and age-dependent effect of HFD and TRF on serum EV profiling

**Figure 1:**
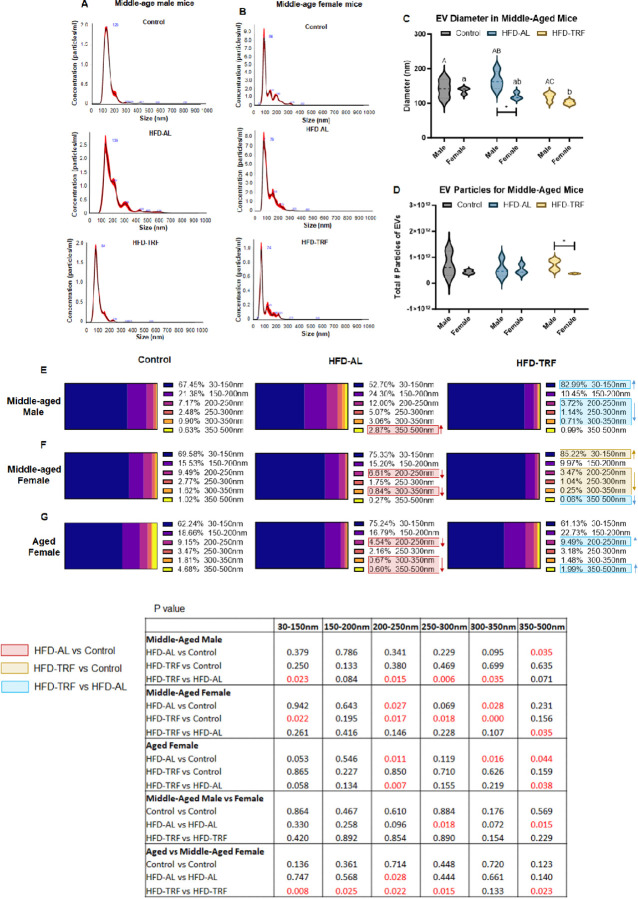
Effect of HFD and TRF on serum EV profiling in middle-aged male and female mice. **A)** Nanoparticle tracking analysis plots for middle-age male mice. **B)** Nanoparticle tracking analysis plots for middle-age female mice. **C)** Violin plot depicting the distribution of serum EV diameter in middle-aged female (n=3) and male mice (n=4). Two-way ANOVA revealed significance between sex and diet type. **D)** Violin plot illustrating the distribution of the average total number of particles of EVs per mL for middle-aged females (n=3) and males (n=4). Two-way ANOVA only showed significance between sex. Student t-test was employed for comparisons between groups. Capitalized letters indicate statistical significance between the experimental groups of male mice, whereas lowercase indicates differences within the female mice group. Different letters indicate statistical significance. *: Indicates statistical significance between the male and female mice. **E)** serum EV size distribution of middle-aged male mice. **F)** Middle-aged female experimental group EV distribution. **G)** Old female experimental group EV distribution. P-values for statistical analysis provided. For the EV size distribution **(E-G)**, two-way ANOVA showed significance between sex and age. Student t-test was employed for comparisons between groups.

Nanoparticle tracking analysis (NTA) revealed that the mean diameter of serum EVs shifted to the right after 28 weeks of HFD feeding compared to the control group in middle-aged male mice; TRF during the last 10 weeks of HFD feeding was able to reverse this shift (Fig. 1A). However, this HFD-induced shift in EV size was not observed in female mice (Fig. 1B). Overall, the average EV size and particle amount were not significantly different in the control group between middle-aged male and female mice. However, the sex difference became evident upon HFD-AL or HFD-TRF. The data indicated that male mice displayed a wider range of EV diameters than female mice under both control and HFD conditions (Fig. 1C), while TRF reduced the size range of EV diameters in both male and females (Fig. 1C). This suggests that serum EVs from middle-aged male mice exhibited greater heterogeneity in size compared to those from female mice. Quantitatively, the mean diameter of EVs in male mice showed a trend towards an increase upon the HFD feeding compared to control mice (Fig. 1C). The HFD group had larger size by 37.9nm (P < 0.02) compared to the TRF mice, indicating that TRF significantly reduced the mean diameter of serum EVs as well as EV size heterogeneity in male mice (Fig. 1C). However, female middle-aged mice had a decreased mean diameter EV in the HFD group by 15.9nm (P = 0.107) compared to the control mice (Fig. 1C). The mean diameter for the TRF female mice was further decreased by 18.6m (P = 0.053) compared to the HFD mice; however, there was a significant difference between the control and TRF female mice (P < 0.006) (Fig. 1C). Additionally, we observed a significant difference in EV size between the middle-aged male and female HFD groups, with the female mice showing a 45.3nm smaller mean size (P < 0.038) (Fig. 1C). There was no alteration by HFD or TRF in the overall particle amount per mL of serum EVs when compared to the control group in both male and female middle-aged mice (Fig. 1D). However, a significant difference in the particle amount was observed between the male and female TRF groups, with the female mice showing lower amount (P < 0.042) (Fig. 1D).

For the age-dependent effect, EVs were isolated from the serum of old female mice (17–21 months old) and compared with the middle-aged female mice (3 months old). We found that TRF could reverse the HFD-induced left shift of EV size distribution (Supplementary Fig. 1A). The mean diameter of EVs was not significantly altered by HFD feeding or TRF in middle-aged female mice (Supplementary Fig. 1B). However, the range of EV diameters was wider in old females in the control condition (Supplementary Fig. 1B), suggesting that old female mice have greater EV size heterogeneity than middle-aged females. When comparing mean diameter size between the old and middle-aged female mice, only the TRF groups showed statistical significance, with the old female mice increased by 50.1nm (P < 0.01) (Supplementary Figure B). Moreover, there was no alteration by HFD or TRF in the overall particle amount per mL of serum EVs when compared to the control group within the old female mice. However, there was statistical significance in the particle amount between the old and middle-aged female mice for the control groups (P < 0.001), HFD groups (P < 0.03), and TRF groups (P < 0.01), with old female mice showing a decrease in each experimental group (Supplementary Fig. 1C). These results indicate that EV secretion declines while EV heterogeneity increases with age, and age influences the effect of HFD and TRF on EV size profiling in female mice.

### Age- and sex-dependent alterations of EV size distribution by HFD and TRF

To identify size-specific EV populations altered by HFD and TRF, we calculated the percentage of different size-based EV populations within the total isolated EVs in each group. The HFD group had a significantly decreased percentage of exosome-sized vesicles (30–150nm) but an increased percentage of larger EVs compared to the control group in middle-aged male mice (Fig. 1E). TRF reversed these changes, leading to a significant increase in the percentage of exosome when compared with both the control group and the HFD group. The TRF group also showed a significantly decreased number of larger EVs (200–350nm) compared to the HFD group (Fig. 1E). The percentage of exosome-sized particles (30–150nm) were increased in the HFD-TRF mice by 30%, whereas the population between 200–350nm were decreased by 15% compared to the HFD-AL mice.

For the middle-aged female mice, the TRF group had a significant increase in the percentage of exosome sized vesicles (30–150nm) when compared with the control group by 36% (Fig. 1F). Mice in the HFD group showed significantly decreased number of larger EVs (200–250nm and 300–350nm) compared to the control (Fig. 1F). Mice in the TRF group also displayed a significantly decreased number of larger EVs (200–350nm) compared to the control (Fig. 1F). Significance was observed only between the HFD groups of middle-aged male and female mice for the 250–300nm and 350–500nm size populations.

In terms of the impact of age on the size distribution of EVs, the HFD group exhibited increased exosome sized vesicles (30–150nm) compared to the control group by 12% and a significantly decreased number of larger EVs (200–250nm and 300–500nm) in old females (Fig. 1G). The TRF group had decreased exosome-sized vesicles (30–150nm) compared to the HFD group by 13% and an increased number of larger EVs (200–250nm and 350–500nm). Interestingly, the significant difference between old female and middle-aged female mice was found in the TRF groups for all of the EV size populations besides the 300–350nm group (Fig. 1F and 1G). For instance, old females had lower percentage of small exosomes (30–150nm) but higher percentage of larger EVs compared to middle-aged females. These results collectively suggest that the effect of HFD and TRF vary by sex and age.

### Characterization of EV protein profiles in young male mice with acute LPS treatment

**Figure 2:**
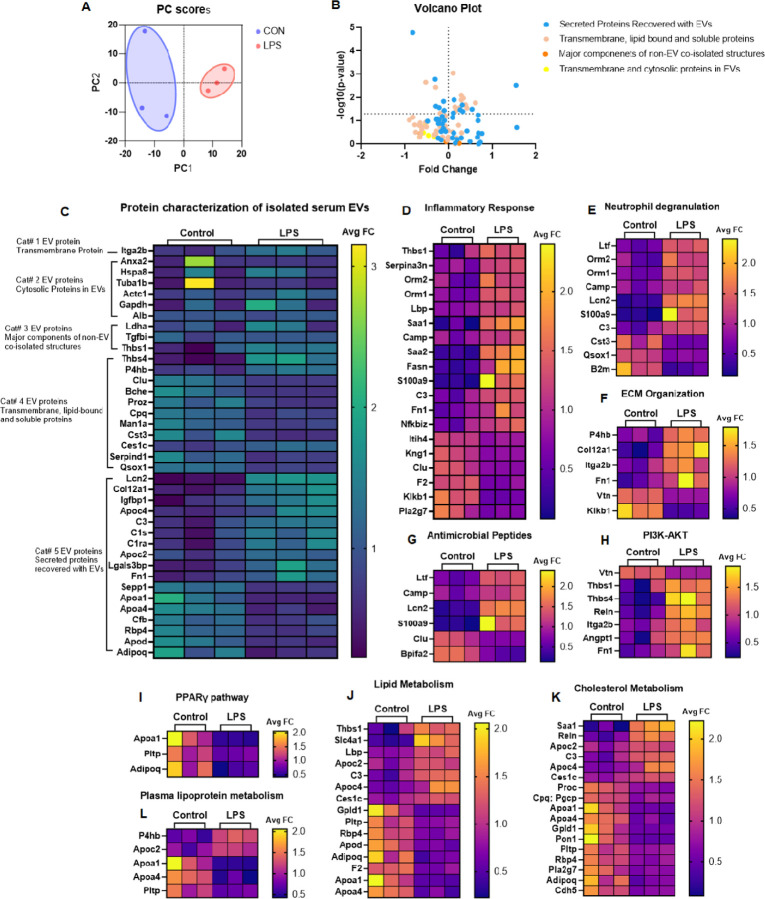
Proteomics characterization of serum EV proteins in young mice. **A)** Scatter plot of the first two principal components (PC1 and PC2) derived from PCA of EV proteins in LPS (n = 3) and control (n = 3) groups. **B)** Volcano plot depicting EV protein characterization. **C)** Heatmap depicting EV protein characterization. **D-K)** Heatmaps depicting alterations of EV proteins in multiple pathways. For all heatmaps, the average fold change (Avg FC) of each protein was calculated as the ratio of its abundance to the average abundance across all control and LPS-treated groups.

To understand how alterations in EV size reflect changes in EV functionalities, we conducted a quantitative proteomics analysis of EV protein composition using the TMT 16plex technique. We characterized serum EV protein profiles in young male mice at 2 months old and determined how acute lipopolysaccharides **(**LPS) treatment for 6 hours affected EV protein profiles. We detected 405 proteins with one peptide match and 287 proteins with two peptide matches. Principal component analysis (PCA) revealed that the protein abundance was specifically clustered by experimental group (Fig. 2A), suggesting that 6-hour LPS treatment can induce significant changes in EV protein profiles. To validate the representation of EVs, we characterized the EV protein composition following the guidelines outlined in the minimal information for studies of EVs (MISEV2023) ^27^. As depicted in the volcano plot (Fig. 2B) and heatmap (Fig. 2C), we detected one protein, Itga2b, belonging to category 1 transmembrane protein, six category 2 cytosolic proteins, three category 3 non-EV proteins (major components non-EV co-isolated structures), eleven category 4 transmembrane, lipid-bound and soluble proteins, and seventeen category 5 secreted proteins recovered with EVs. Interestingly, acute LPS treatment significantly altered the abundance of category 4 and 5, but not category 1–3 proteins. These results indicate that the serum particles isolated have good representation of EVs. Furthermore, Metascape (https://metascape.org) analysis showed the enriched ontology clusters (pathways) involving EV proteins altered by LPS in Supplementary Fig. 2A and 2B. Ten protein-protein interaction (PPI) networks (MCODE components) involving EV proteins were identified, including the complement and coagulation cascade, extracellular matrix (ECM) organization, neutrophil degranulation, platelet activation, amyloid fiber formation, cytokine signaling in immune system, respiratory electron transport, VEGFA/VEGFR2 signaling, etc. (Supplementary Fig. 2C). Acute LPS treatment affected most of the MCODE components except for intermediate filament organization, keratinization, and respiratory electron transport (Supplementary Fig. 2D). Moreover, we identified 5 PPI networks of EV proteins specifically altered by LPS, and these PPI networks regulate plasma lipoprotein metabolism, protein-lipid remodeling, amyloid fiber formation, complement and coagulation cascade, platelet activation, and RHO GTPase effectors (Supplementary Fig. 2E). Specifically, EV proteins involved in inflammatory response, neutrophil degranulation, ECM organization, antimicrobial proteins, and PI3K-AKT pathway were upregulated upon LPS treatment (Fig. 2D–2H), whereas EV proteins regulating PPARγ pathway were down-regulated (Fig. 2I). Additionally, LPS-induced alterations in EV proteins include lipid metabolism, cholesterol metabolism, and plasma lipoprotein metabolism (Fig. 2J–2L). These results suggest that serum EVs can acutely respond to LPS and EV proteins reflects LPS-induced inflammatory responses.

### Identification of serum EV protein profiles and functions affected by HFD and TRF in middle-age male mice

**Figure 3:**
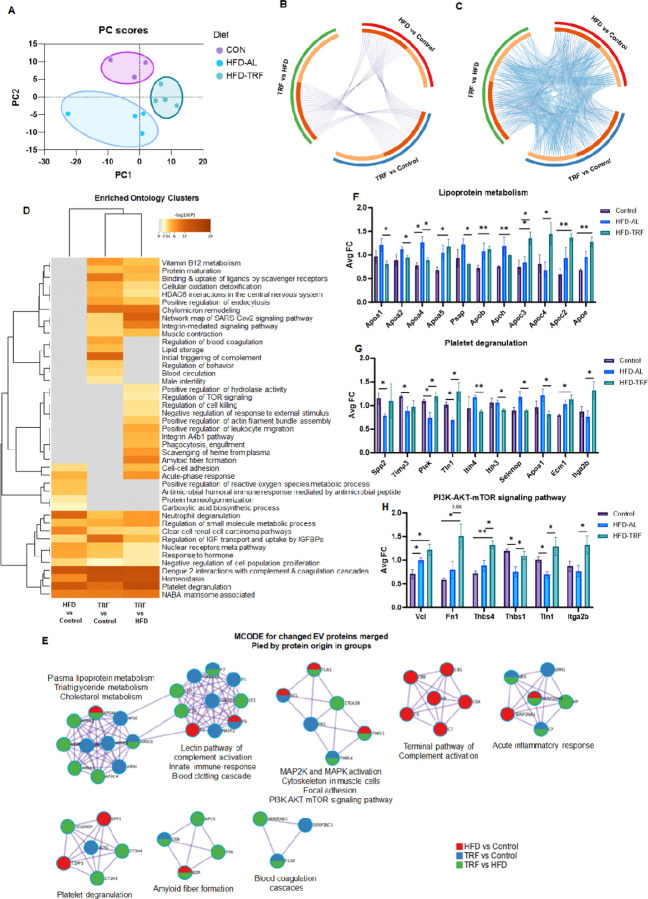
Effect of HFD and TRF on serum EV protein profiles in middle-aged male mice. **A)** Scatter plot of the first two principal components (PC1 and PC2) derived from the PCA of EV proteins in control (n = 3), HFD-AL (n = 4), and HFD-TRF (n = 4) groups. **B)** Circos plot showing overlap of altered EV proteins from three group comparisons. **C)** Circos plot showing shared enriched ontologies of altered EV proteins from three group comparisons. **D)** Enriched ontology clusters of altered EV proteins from three group comparisons. **E)** Protein-protein interaction network of altered EV proteins merged and pied by three group comparisons.**F-H)** The abundance of altered EV proteins in their respective clusters or pathways. **Note:** For the Circos plots (B and C), on the outside, each arc represents the identity of each EV protein list. On the inside, each arc represents an EV protein list, where each protein member of that list is assigned a spot on the arc. Dark orange color represents the proteins that are shared by multiple lists and light orange color represents proteins that are unique to that protein list. Purple lines link the same protein that are shared by multiple protein lists. The greater the number of purple links and the longer the dark orange arcs implies greater overlap among the input protein lists.

A quantitative proteomics analysis on serum EVs of middle-aged male mice identified 358 proteins with one peptide match and 231 proteins with two peptide matches. Figure 3A illustrated the PCA on the normalized abundance of 358 proteins with one peptide match for all three experimental groups. The PCA showed that the specific protein abundance was separated by experimental group, with no overlap between the three groups, suggesting that EVs from the three different conditions possess a unique set of proteins. Next, we compared protein abundance among three groups to identify EV proteins with differential abundance for further GO analysis. The Circos plot from gene overlap analysis of individual altered EV proteins showed that EV proteins altered by TRF (TRF vs Control) only partially overlapped with those changed by HFD (HFD vs Control) (Fig. 3B). However, the functional overlap analysis of shared enriched ontologies for altered proteins from the three comparisons (HFD vs Control, TRF vs Control, and TRF vs HFD) indicated significant functional overlap between the HFD and TRF groups (Fig. 3C), suggesting that TRF could reverse the same biological processes altered by HFD, but not directly the same sets of proteins. Enriched ontology clusters of altered EV proteins demonstrated that EVs are involved various biological processes, and these functions can be significantly changed by dietary interventions (HFD and TRF) (Fig. 3D). Among all EV proteins changed by HFD and/or TRF, 7 PPI networks were identified (Fig. 3E). When changes were merged and analyzed by three group comparisons, it clearly showed that PPI networks were specifically and differentially affected by HFD and TRF (Fig. 3E). For instance, TRF uniquely affected networks unaltered by HFD, such as lipoprotein metabolism, blood coagulation, and amyloid fiber formation, but had no effect on the terminal pathway of complement (Fig. 3E). Intriguingly, TRF significantly upregulated apoproteins including Apoa5, Apob, Apoh, Apoc3, Apoa4, Apoc2, and Apoe (Fig. 3F), and restored most of network components involved in platelet activation (Fig. 3G). Lastly, TRF was able to significantly activate PPI components of PI3K AKT-mTOR signaling pathway (Fig. 3H).

### Gene Set Enrichment Analysis of EV proteins altered byHFD and TRF

**Figure 4:**
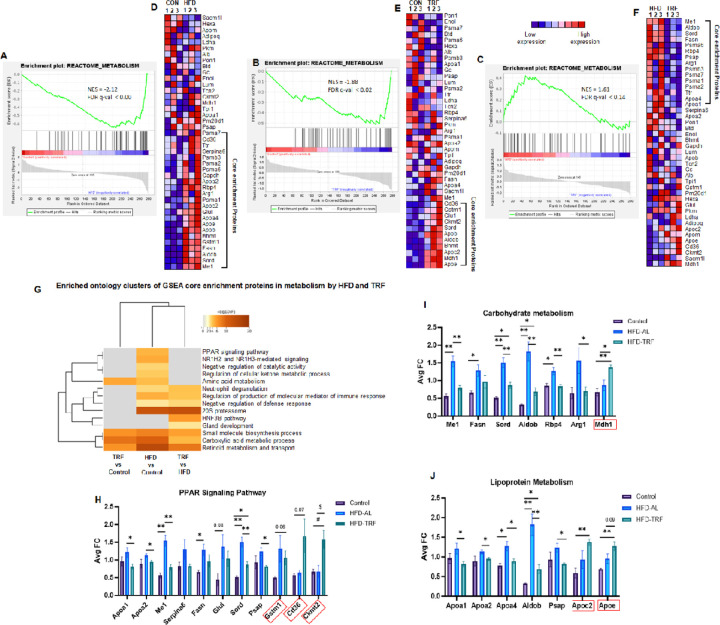
GSEA of altered serum EV proteins in metabolism by HFD and TRF. **A)** Enrichment score plot of metabolism pathways for group comparisons of EV protein abundance between control and HFD-EVs. **B)** Enrichment score plot of metabolism pathways for group comparisons of EV protein abundance between control and TRF-EVs. **C)** Enrichment score plot of metabolism pathways for group comparisons of EV protein abundance between HFD-EVs and TRF-EVs. **D-F)** Corresponding heatmaps of EV protein abundance to the enrichment score plots. **G)** Enriched ontology clusters of altered EV proteins from three group comparisons. **H-J)** The abundance of altered EV proteins in their respective clusters or pathways. Note: The degree of enrichment is indicated on the figures by a normalized enrichment score (NES) **(A-C)**. A significant positive NES value indicates that proteins in the enrichment core tend to appear towards the top of the protein set whereas a significant negative NES indicates the opposite, towards the bottom of the protein set as shown in the heatmaps **(D-F)**.

Similar to Metascape analysis, Gene Set Enrichment Analysis (GSEA) identified significant pathways, including metabolism, the innate immune system, the complement system, neutrophil degranulation, developmental biology, and others. The NES (normalized enrichment score) in Fig. 4A–4B shows the enrichment core proteins were upregulated in the HFD-EVs (−2.12) and TRF-EVs (−1.88) compared to the control-EVs. The NES score for HFD-EVs versus TRF-EVs (1.61) indicates the core-enriched proteins were down-regulated in the TRF-EVs compared to HFD-EVs. Proteins identified via GSEA to be within the metabolism pathway are shown in Figs. 4D–4F, with the core enriched proteins bracketed. The 23 core enrichment proteins were upregulated in the HFD-EVs versus control-EVs (Figs. 4D). When comparing TRF-EVs versus HFD-EVs, 15 out of the 23 HFD-upregulated core enrichment proteins were downregulated by TRF (Fig. 4F). Further Metascape analysis clustered these core proteins into specific metabolic pathways (Fig. 4G). As the top cluster identified, components of PPAR signaling pathway were significantly altered by HFD and reversed by TRF (Fig. 4H). Additionally, two specific sets of proteins involved in carbohydrate metabolism and lipid metabolism were significantly upregulated by HFD and downregulated by TRF (Fig. 4I and 4J). Six EV proteins (Gstm1, Cd36, Ckmt2, Mdh1, Apoc2, and Apoe) were significantly upregulated by TRF in the PPAR signaling pathway, carbohydrate metabolism, and lipid metabolism (Fig. 4H–4J). This upregulation response is unique to TRF, as the abundance of these proteins was not affected by HFD.

**Figure 5:**
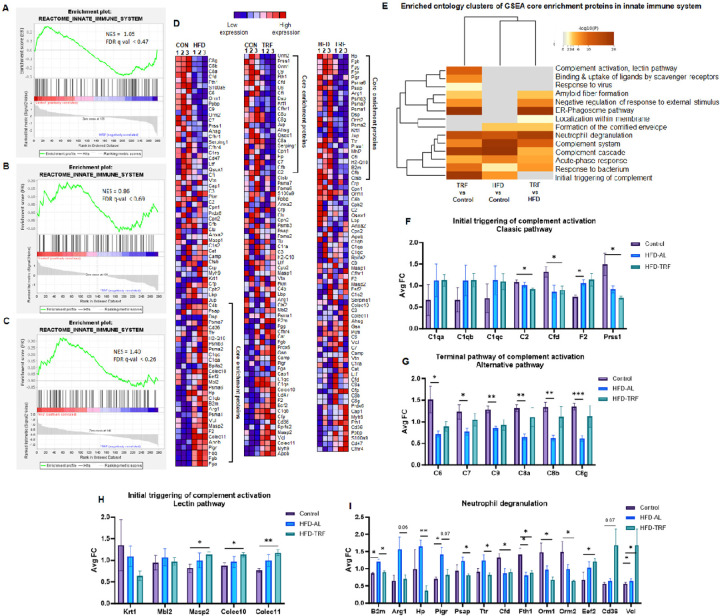
GSEA of altered serum EV proteins in innate immune system by HFD and TRF. **A)** Enrichment score plot of abundance of EV proteins involved in innate immune system pathway for group comparisons between control and HFD-EVs. **B)** Enrichment score plot of innate immune system pathway for group comparisons of EV protein abundance between control and TRF-EVs. **C)** Enrichment score plot of innate immune system pathway for group comparisons of EV protein abundance between HFD-EVs and TRF-EVs. **D)** Corresponding heatmaps of EV protein abundance to the enrichment score plots.**E)** Enriched ontology clusters of altered EV proteins from three group comparisons. **F-I)** The abundance of altered EV proteins in their respective clusters or pathways.

For the innate immune system, the NES value of −1.05 (CON vs HFD) indicates that 30 core enriched proteins in immune system were upregulated in the HFD-EVs compared to control-EVs (Figs. 5A and 5D). The core enriched proteins for CON vs TRF were downregulated in the TRF-EVs (Fig. 5B and 5D), the downregulation of the same set of EV proteins was also observed in HFD-EVs versus Control-EVs (Fig. 5D). However, when comparing HFD-EVs and TRF-EVs, the core enriched proteins were down-regulated in the TRF-EVs compared to HFD-EVs (Fig. 5D). To delve deeper into the immune pathway-specific changes, we performed Metascape analysis on core enrichment proteins found by GSEA in innate immune system from the three group comparisons. As shown in Fig. 5E, these core enrichment proteins fell into the following main categories: lectin pathway of complement activation, neutrophil degranulation, and initial triggering of complement, and terminal pathway of complement activation. HFD had no impact on initial triggering of classic complement pathway (Fig. 5F). However, HFD significantly downregulated the terminal pathway of alternative complement activation (Fig. 5G), and this downregulation was partially prevented by TRF (Fig. 5G). Moreover, TRF significantly upregulated proteins involved in lectin pathway of complement activation such as Masp2, Colec10, and Colec11 (Fig. 5H). More intriguingly, TRF extensively affected neutrophil degranulation pathway by directly reversing HFD-induced changes as well as up- or down-regulating unique sets of proteins in the neutrophil degranulation pathway, independent of HFD-induced changes (Fig. 5I). Collectively, these results clearly demonstrate that HFD affects significant pathways and biological processes, while TRF reverses these functional processes rather than the same individual proteins.

Additionally, GSEA identified other three pathways to be significant, including developmental biology, post translation protein modification, and transport of small molecules pathways as indicated in Supplementary Figs. 3–5. For the developmental biology (Supplementary Fig. 3A–3F), a large set of core enrichment proteins were found to be downregulated in both the TRF-EVs versus the control-EVs (Supplementary Fig. 3B and 3E) and the TRF-EVs versus the HFD-EVs (Supplementary Fig. 3C and 3F). Most of the core enrichment proteins are keratins known as biomarkers of DNA damage and tumorigenesis. For instance, Keratin 17 can be induced in response to DNA damage^28^ and robustly up regulated in multiple types of epithelial tumors^29^ and upregulation of Keratin 17 correlates with poor prognoses for some cancers ^30–34^.

## DISCUSSION

A previous study showed an increase in the number of circulating EVs in a HFD-induced obesity ^35^. This increase in a HFD state suggests a role of EVs in intercellular mediation due to metabolic disturbances. In this study, our results indicate that a HFD led to an increase in mean diameter of serum EVs compared to the control, which could be reversed by TRF in male mice. However, this HFD-induced change was not present in female mice, indicating a different metabolic response to HFD between sexes. Although TRF intervention could reduce both male and female mean diameter of EVs, there was a notable difference in male and female TRF groups with the females displaying lower particle amount. These findings indicate there is an impact between sex and dietary factors in altering serum EV size and secretion. Furthermore, HFD led to a decrease in the percentage of serum exosome-sized vesicles but an increase in larger EVs compared to the control in middle-aged male mice. Interestingly, TRF could increase the percentage of specific exosome-sized vesicles (30–150nm) consistently in both middle-aged male and female mice, suggesting a relationship between small EVs and TRF improvement of metabolic health.

We have demonstrated that there was a significant difference in the mean EV diameter between middle-aged and aged female mice in the TRF groups, with the aged female mice having an increased mean diameter versus the middle-aged female mice, indicating an age-dependent effect in response to TRF. This is different from a previous study published, which indicated age decreased the average diameter in older mice compared to young female mice. However, the middle-aged female mice in this study were 8 months old, which could be why no significant difference was seen between the two groups ^21^. Although no significant alterations were seen in the overall particle concentration of serum EVs within aged female mice across the experimental groups, a significant decrease in particle amount was observed in aged female compared to middle-aged female across all experimental groups, suggesting an age-related decline in EV secretion which is consistent with a previous study ^21^. These findings indicate age has an influence on EV size, secretion, and the responsiveness to dietary intervention in female mice.

The quantitative proteomics analysis indicated that acute inflammatory stimulation via LPS could notably modify EV protein composition and functions, reflecting the impact of LPS on inflammatory response, lipid, and cholesterol metabolism, the PPARγ pathway, and ECM organization. Both PCA and Volcano plots further highlighted the differential abundance of proteins caused by HFD and TRF, indicating complex molecule changes with EVs under the different dietary factors. GO analysis identified pathways impacted by both TRF and HFD, including complement activation, blood coagulation, metabolism, and immune system regulation. Metascape analysis results demonstrate that a low number of EV proteins altered due to HFD are reversed directly by TRF. However, the biological processes and pathways associated with altered EV proteins highly overlap across the three group comparisons, and HFD, TRF, and LPS affect specific PPI networks. GSEA and Metascape analysis identified specific sets of EV proteins regulating multiple pathways of metabolism and immune responses across the three group comparisons. Among these pathways, the two key metabolic regulatory pathways, the PPAR signaling pathway and the AKT-mTOR signaling pathway, as well as innate immune system pathways like the complement cascade and neutrophil activation, are highly responsive to HFD, TRF, and LPS. Additionally, we found that TRF could stimulate unique sets of EV proteins that activate the PPAR, AKT-mTOR, and lectin complement activation pathways beyond HFD-induced changes. Specifically, the lipoprotein metabolism/remodeling and carbohydrate metabolism pathways have been significantly altered by HFD, a change that can be restored by TRF. Overall, 23 core enrichment proteins involved in metabolism were upregulated in HFD-EVs, and 65% of them could be reversed to levels comparable to control EVs. These findings suggest that both HFD and TRF affect EV proteins and their associated network functions, reflecting changes in metabolism. EV proteins have potential as biomarkers and can be developed to assess EV functions, metabolic responses to dietary intervention, and disease conditions. Furthermore, in the developmental biology pathway, TRF was able to significantly down-regulate Keratins, which are known biomarkers of DNA damage and tumorigenesis. This suggests that TRF may have benefits in reducing aging and the risk of tumorigenesis.

The alterations in the innate immune system pathway are somewhat complex. There were two sets of core enrichment proteins that were differentially regulated in HFD-EVs and TRF-EVs. Further analysis on GSEA core enriched proteins using Metascape indicates that HFD primarily affects the terminal alternative complement pathway and initial classic complement pathway, but not the lectin complement pathway. HFD significantly reduces the terminal alternative complement pathway activation, which can be partially restored by TRF, although TRF is unable to restore classic complement pathway activation. Interestingly, TRF significantly upregulates the components of the lectin pathway. Lastly, when looking at neutrophil degranulation, TRF effectively reverse the HFD-induced alterations in EV proteins within an enriched neutrophil degranulation cluster, suggesting a restoration of the neutrophil degranulation pathway in TRF-EVs.

Overall, these findings highlight the intricate interplay between dietary interventions, EV protein composition, and functional pathways, suggesting that TRF may be useful in mitigating HFD-induced alterations in EV functions and their roles in metabolism and immune system regulation. Our results also indicate that each group or population of EVs contains unique sets of proteins that exert specific biological functions. These findings contribute to a deeper understanding of the role of EVs in mediating physiological responses to dietary interventions and their potential as therapeutic strategies for metabolic health.

## Supplementary Material

Supplement 1

## Data Availability

Data will be made available on request.
