# Supplementary material for: Serum Extracellular Vesicles Reveal Metabolic Responses to Time-Restricted Feeding in High Fat Diet-Induced Obesity in Male Mice
Source: Res Sq. 2024 Sep 24:rs.3.rs-4745029. Preprint. [Version 1] doi: 10.21203/rs.3.rs-4745029/v1 (PMC11469403; doi:10.21203/rs.3.rs-4745029/v1)
Supplement: Supplement 1 [file NIHPPrs4745029v1-supplement-1.pdf]

Supplementary Files

This is a list of supplementary files associated with this preprint. Click to download.

- EVmanuscriptSupplementaryFileIntOfObesityJuly152024Finalsubmission.pdf
